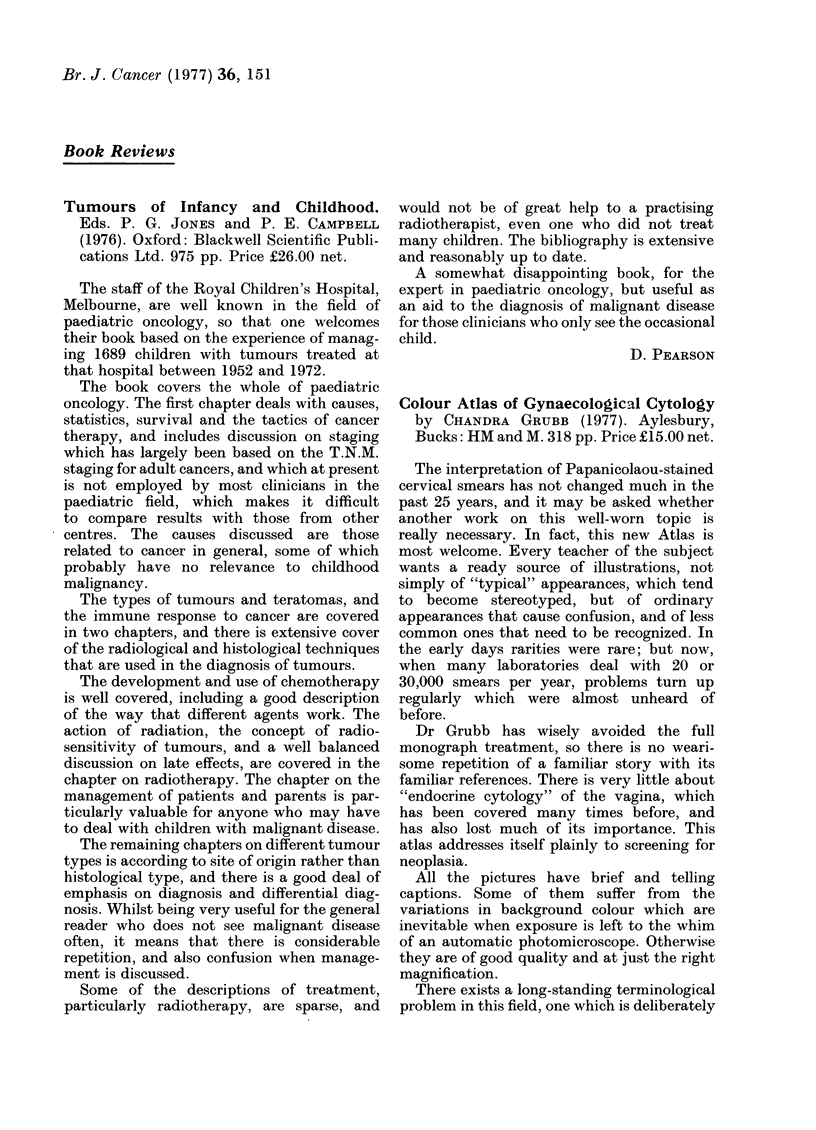# Tumours of Infancy and Childhood

**Published:** 1977-07

**Authors:** D. Pearson


*Br. J. Cancer* (1977) **36**, 151

### *Book Reviews*

**Tumours of Infancy and Childhood.** Eds. P. G. JONES and P. E. CAMPBELL (1976). Oxford: Blackwell Scientific Publications Ltd. 975 pp. Price £26.00 net.

The staff of the Royal Children's Hospital, Melbourne, are well known in the field of paediatric oncology, so that one welcomes their book based on the experience of managing 1689 children with tumours treated at that hospital between 1952 and 1972.

The book covers the whole of paediatric oncology. The first chapter deals with causes, statistics, survival and the tactics of cancer therapy, and includes discussion on staging which has largely been based on the T.N.M. staging for adult cancers, and which at present is not employed by most clinicians in the paediatric field, which makes it difficult to compare results with those from other centres. The causes discussed are those related to cancer in general, some of which probably have no relevance to childhood malignancy.

The types of tumours and teratomas, and the immune response to cancer are covered in two chapters, and there is extensive cover of the radiological and histological techniques that are used in the diagnosis of tumours.

The development and use of chemotherapy is well covered, including a good description of the way that different agents work. The action of radiation, the concept of radiosensitivity of tumours, and a well balanced discussion on late effects, are covered in the chapter on radiotherapy. The chapter on the management of patients and parents is particularly valuable for anyone who may have to deal with children with malignant disease.

The remaining chapters on different tumour types is according to site of origin rather than histological type, and there is a good deal of emphasis on diagnosis and differential diagnosis. Whilst being very useful for the general reader who does not see malignant disease often, it means that there is considerable repetition, and also confusion when management is discussed.

Some of the descriptions of treatment, particularly radiotherapy, are sparse, and would not be of great help to a practising radiotherapist, even one who did not treat many children. The bibliography is extensive and reasonably up to date.

A somewhat disappointing book, for the expert in paediatric oncology, but useful as an aid to the diagnosis of malignant disease for those clinicians who only see the occasional child.

D. PEARSON